# BRAF Mutated Colorectal Cancer: New Treatment Approaches

**DOI:** 10.3390/cancers12061571

**Published:** 2020-06-14

**Authors:** Javier Molina-Cerrillo, María San Román, Javier Pozas, Teresa Alonso-Gordoa, Miguel Pozas, Elisa Conde, Marta Rosas, Enrique Grande, María Laura García-Bermejo, Alfredo Carrato

**Affiliations:** 1 Medical Oncology Department, University Hospital Ramon y Cajal, 28034 Madrid, Spain; mariasanro21@gmail.com (M.S.R.); pozas.javier@gmail.com (J.P.); talonso@oncologiahrc.com (T.A.-G.); poszas.miguel12@gmail.com (M.P.); acarrato@telefonica.net (A.C.); 2 CIBERONC, The Ramón y Cajal Health Research Institute (IRYCIS), 28034 Madrid, Spain; 3 Medicine School, Alcalá University, 28805 Madrid, Spain; 4 Biomarkers and Therapeutic Targets Group and Core Facility, The Ramón y Cajal Health Research Institute (IRYCIS), CIBERONC, 28034 Madrid, Spain; elisa.condem@gmail.com (E.C.); garciabermejo@gmail.com (M.L.G.-B.); 5 Pathology department, University Hospital Ramon y Cajal, 28034 Madrid, Spain; nenautopsias@gmail.com; 6 Department of Medical Oncology, MD Anderson Cancer Center, 28033 Madrid, Spain; egrande@oncomadrid.com

**Keywords:** BRAF, colorectal cancer, tyrosine kinases, immunotherapy, CXCR4

## Abstract

Colon cancer is one of the most frequently diagnosed malignancies in adults, considering both its incidence and prevalence. Anatomically, the right colon is considered as being from the cecum to the splenic flexure, and the left colon is from the splenic flexure to the rectum. Sidedness is a surrogate of a wide spectrum of colorectal cancer (CRC) biology features (embryology, microbiome, methylation, microsatellite instability (MSI), BRAF, aging, KRAS, consensus molecular subtypes (CMS), etc.), which result in prognostic factors. Different molecular subtypes have been identified, according to genomic and transcriptomic criteria. A subgroup harboring a BRAF mutation has been described, and represents approximately 10% of the patients diagnosed with colon cancer. This subgroup has morphological, clinical, and therapeutic characteristics that differ substantially from patients who do not carry this genetic alteration. Unfortunately, there is no established standard of care for this particular cohort of patients. This manuscript aims to study the biology of this subgroup of colon cancer, to understand the current approach in clinical research.

## 1. Introduction

Colorectal cancer (CRC) incidence and mortality rates vary noticeably around the world. Overall, it is the third largest cause of cancer death, and the third most frequently diagnosed tumor, involving 11% of all new cases of cancer worldwide [1]. Although global mortality is decreasing, the subgroup of younger patients (<50 years old) has experienced a growing incidence and mortality rate. Up to one quarter of patients have metastatic colorectal cancer (mCRC) disease at initial diagnosis, and nearly half of those who are candidates for primary tumor surgery will eventually develop metastasis, resulting in a 5-year survival rate of 14% [2].

Rates of CRC are substantially higher among males in comparison with females. In men, it entails the third most common type of cancer diagnosed, and the fourth most common cause of cancer death. Among females, it is the second most commonly diagnosed malignancy, and the third most common cause of cancer death [3].

The highest incidence rates are found in Australia, New Zealand, Europe, and North America, whereas South-Central Asia and Africa report a much lower incidence. In most Western countries, CRC incidence has been stable, or has experienced a slight increase within the last few years. In contrast, areas with a historical low incidence rate of CRC (Spain, Eastern Europe, or Eastern Asia) have experienced a rapid increase over the past few decades [3].

Up to 70% of CRC are sporadic and mainly associated with environmental and dietary factors. Less than 10% of patients with CRC have an inherited predisposition due to several genetic alterations, some of which can be already identified by specific tests. This category is subdivided into polyposis diseases (MUTYH-associated polyposis, familiar adenomatous polyposis, Cowden syndrome, and Peutz-Jeghers syndrome) and non-polyposis diseases, such as Lynch syndrome. Another pattern of presentation is the familial CRC that accounts for up to 25% of all CRC. This subset of patients has an increased risk of developing CRC, without fulfilling the criteria of the above-mentioned syndromes.

## 2. Molecular Pathogenesis of Colorectal Cancer

CRC arises from the accumulation of genetic and epigenetic mutations, which result in the alteration of different molecular pathways. Those changes lead to the transformation of a normal glandular epithelial cell into a benign neoplasm and, later, into an invasive carcinoma. Depending on the germline and somatic mutations burden, there are various mechanisms involved, making CRC a complex and heterogeneous neoplasm. Over the past decades, there has been a great interest to further understand the molecular biology of CRC, which has allowed the development of targeted therapies that are nowadays incorporated into daily clinical practice, in parallel with the implementation of routine genetic testing, such as the detection of RAS or BRAF mutations. 

Most human CRCs are believed to arise from benign lesions, usually adenomatous polyps [4], due to a disruption in the mechanisms regulating epithelial renewal. There are exogenous factors, such as diet, smoking, alcohol consumption, obesity, or sedentary lifestyle, and endogenous factors, such as oxidative stress, chronic inflammation, or intestinal dysbiosis, which lead to the transformation of benign adenomatous polyps into a dysplastic polyp, favoring cumulative mutations, which finally lead to the development of CRC [5]. Several data support this hypothesis; firstly, in animal models, carcinoma arise mainly from benign polyps; secondly, areas of adenomatous tissue can be found within the limits of CRCs; thirdly, benign and malignant tumors are found in the same distribution with a time lapse of 10 to 15 years; and lastly, it has been proven that the removal of premalignant polyps reduces the incidence of CRC [6]. 

In contrast to the conventional adenoma-carcinoma pathway, there is evidence that serrated polyps may constitute an alternative pathway to CRC carcinogenesis, accounting for up to 20% of them [7]. Clinical differences between adenoma and serrated polyps are sidedness (serrated are more frequently located in the left side), size (serrated used to be larger), and malignant potential (higher in the case of serrated polyps). Histological characterization of serrated polyps allows to categorize them into three subtypes: traditional serrated adenomas (TSA), mixed serrated polyps (MP) and sessile serrated adenomas (SSA). From a molecular point of view, serrated lesions seldom present APC mutations. The majority of CRCs arising from serrated polyps tend to carry BRAF mutations, while KRAS mutations are more infrequent. Microsatellite instability (MSI) and CpG island methylator phenotype (CIMP) represent the two main molecular pathways implicated in the transformation of benign serrated polyps into CRC [8]. 

Several molecular pathways have been associated with CRC tumorigenesis and the most significant ones are exposed below.

### Molecular Pathways Involved in CRC Carcinogenesis

The majority of CRCs develop through the chromosomal instability pathway (CIN), which is defined as the presence of gross structural aberrations such as deletions, insertions, and loss of heterozygosity, results in an altered karyotype [5,7]. The most important genes involved in these chromosome alterations are APC (5q deletion), TP53 (17p deletion), and DCC/MADH2/MADH4 (18q deletion) [9]. APC is a tumor suppressor gene, which plays a key role in the earliest step of CRC carcinogenesis. It encodes the APC protein, which is the main component of the B-catenin destruction complex involved in the suppression of the Wnt/β-catenin signaling pathway [10]. The loss of function of the APC protein prevents the formation of this complex, resulting in the accumulation of β-catenin protein in the cytoplasm, later on being translocated to the nucleus, where it binds to the TCF/LEF transcription factors. This binding enhances the overactivation of the Wnt signaling pathway, hence, promoting cell proliferation, migration, invasion, and metastasis [11]. Although CIN represents the main molecular pathway involved in sporadic CRCs, it is also observed in familial adenomatous polyposis (FAP), due to a germline mutation of the APC gene, with a 100% risk of developing CRC within the patient’s life-span [12]. 

The mismatch repair (MMR) pathway is based on mutations or epigenetic phenomena in one of the DNA MMR genes (most commonly MLH1, MSH2, MSH6 and PMS2), which leads to the dysfunction of the DNA MMR enzymes [13]. Consequently, there is an accumulation of DNA abnormalities, particularly within mononucleotide or dinucleotide repeats found throughout the genome, which are known as microsatellites. Since the DNA damage fails to be repaired, there is a change in the size of microsatellites, which can promote genes deletions or insertions (InDel) and, accordingly, the proteins encoded by them [14]. Moreover, InDel induces frameshift and the production of truncated/altered proteins and, therefore, an increase in neoantigens expression. This is one of the reasons why MSI tumors are more immunogenic than others [7,15].

On the one hand, in hereditary CRC, which represents less than 5% of all CRC diagnosed, the MMR pathway is involved in the development of more than 95% of patients with Lynch syndrome. The other less than 5% may be explained by alterations in proofreading domain of POLE and POLD1 genes [16]. On the other hand, in sporadic CRC, about 20% harbor MSI, due to the hypermethylation of MLH1 [15]. Interestingly, sporadic high microsatellite instability CRC is associated with a higher prevalence of BRAF mutations compared with microsatellite stable (MSS) tumors or hereditary nonpolyposis CRC [17]. There are some histologic and clinical features associated with CRCs developed through these pathways that are worth commenting upon. Firstly, the rate of adenoma-to-carcinoma progression tends to evolve more quickly in MSI tumors, when compared to MSS tumors. This is remarkable, particularly in the serrated pathway data reported in screening programs. [18]. Additionally, CRC with MSI-driven tumorigenesis usually have an increased mucin production, which is a particular feature that distinguishes them from other CRCs.

The CIMP pathway is based on epigenetic instability, and it is characterized by a vast hypermethylation of the promoter region of MMR enzymes, such as MLH1, which is abundant in CpG islands. This fact will probably result in chromosomal instability, with frequent loss of heterozygosity at key tumor suppressor genes [19]. These alterations have been identified in approximately 20–35% of CRC, and are characteristic of the serrated polyps that progress to CRC [20]. A panel of methylation markers is currently being developed, in order to classify CRC according to low or high levels of DNA methylation [21]. CIMP-high tumors have a distinct profile, being associated with the proximal colon location, poor histologic differentiation, MSI status, BRAF mutations and wild-type KRAS [22]. Conversely, CIMP-low tumors are linked to a high rate of KRAS and TP53 mutations (92% and 71%, respectively) [23,24]. 

## 3. Consensus Molecular Subtypes

In 2015, Guinney et al. analyzed tumor samples from 4151 patients with stage II and III CRC, and used a network-based approach to identify four consensus molecular subtypes (CMS), demonstrating independent prognostic value, and significant association with clinical and biological features [25].

The CMS1 category is also referred to as “MSI immune”, and represents approximately 14% of CRC. It has a high rate of mutations and low prevalence of somatic copy number alterations (SCNA). Most MSI tumors belong to this subgroup, showing a deficient DNA mismatch repair machinery. They also display a hypermethylation status, resulting in a CIMP+ phenotype. As expected, mutations in BRAF are frequently found. In addition, there are some specificities regarding the biological behavior of these tumors, particularly an increased expression of genes involved in immune infiltration, mainly composed of type-1 helper and cytotoxic T lymphocytes, as well as an upregulation of proteins participating in immune response pathways. From a clinical point of view, CMS1 tumors are more common in females and in the right colon location, and they tend to present themselves with higher histopathological grade, hence, resulting in a poorer prognosis when compared to other molecular subtypes. This is in accordance with the available data coming from clinical trials where they analyze patients harboring MSI and BRAF-mutated tumors [17,25]. The association between CMS and microbiome has also been studied, finding a high population of Fusobacterium hwasookii and Porphyromonas gingivalis in CMS1 tumors [26]. In the metastatic setting, considering the strong immunogenicity of these tumors, the use of checkpoint inhibitors is a novel therapeutic approach in MSI CRC. Indeed nivolumab (anti PD1) [27], pembrolizumab (anti PD1) [28] and the combination of nivolumab and ipilimumab (anti CTLA4) [27] have been approved for this subset of patients. A retrospective analysis of the Alliance for Clinical Trials in Oncology suggests that patients with CMS1 tumors may benefit more from bevacizumab (anti-VEGF), when compared to anti-EGFR targeted agents, such as cetuximab [29].

CMS2 tumors are also known as “canonical”, and they account for 37% of CRC. In contrast to the CMS1 category, they have a high prevalence of SCNAs, thus tending to display a CIN phenotype. Additionally, CMS2 CRC usually present an epithelial differentiation along with an upregulation of Wnt and MYC, which are known to be involved in carcinogenesis. As opposed to CMS1, these tumors are mainly left-sided, and they are associated with a longer survival after relapse [25]. From a therapeutic point of view, a retrospective analysis of the NSABP C-07 trial showed that a subset of CMS2 patients benefit from the use of oxaliplatin [30]. It has also been reported that CMS2 CRC are ineffective in establishing patient-derived xenografts (PDXs). Prasetyanti et al. suggest that CMS2 tumors were difficult for PDX achievement, and that those which successfully established PDX had a poorer prognosis, thus, being able to distinguish two different outcome subgroups within this category of CRC [31]. Similar to CMS1, an association with microbiome was also found, CMS2 tumors are enriched with Selenomas and Prevotella species [26]. 

Roughly 13% of CRC belong to CMS3, which are also called “metabolic” tumors. Despite displaying a CIN phenotype, CMS3 tumors carry a distinctive genomic profile, namely lower prevalence of SCNAs, lower hypermethylation when compared to CMS1, variable MSI and low CIMP status. Additionally, KRAS mutation is more prevalent within this subgroup [25]. KRAS-mutated tumors have a limited treatment approach since they have a poor response to anti-EGFR therapy. Nonetheless, the use of cetuximab in CMS3 KRAS wild-type tumors may prove to be useful. A detailed analysis of the AGITG MAX clinical trial suggested that CMS2 and CMS3 patients may benefit from the addition of anti-VEGF bevacizumab to first line capecitabine-based chemotherapy [32]. Other potential therapeutic strategies, such as targeting TKR, HER-2, or CBS, are under clinical research. 

Finally, CMS4 tumors are also known as “mesenchymal”. They stand out for exhibiting extremely high SCNAs, along with low levels of hypermutation and MSS status. The genomic profile displayed by these tumors are consistent with the presence of an active stroma: the upregulation of genes involved in epithelial mesenchymal transition (EMT), as well as signatures associated with the activation of transforming growth factor β (TGF β) signaling, complement inflammatory system, angiogenesis, and matrix remodeling pathways [25]. All of this creates a pro-inflammatory environment, with the recruitment of regulatory T cells, myeloid-derived suppressor cells, and macrophages. The presence of IL-23 and IL-17 leads to a higher rate of colitis-related CRC in this subgroup of patients [33]. In terms of survival, CMS4 metastatic CRCs have the worst 5-year overall survival (OS), and progression free survival (PFS), when compared to the rest of subtypes. A recent analysis of the FIRE3 trial showed that, in contrast with CMS1, patients with CMS4 CRC possibly benefit more from adding cetuximab, instead of bevacizumab to first line treatment with FOLFIRI (5-fluorouracil + irinotecan) [34].

## 4. BRAF Mutations in Colorectal Cancers

**Figure 1:**
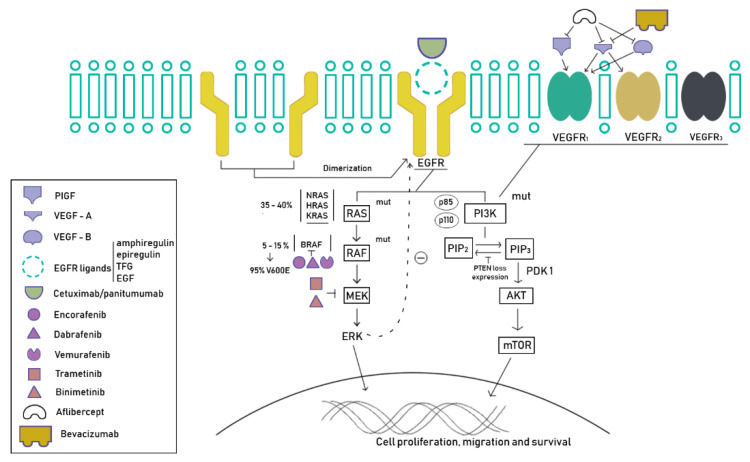
EGFR (epidermal growth factor receptor) and VEGFR (vascular endothelial growth factor receptor) signalization pathways. BRAF plays a central role in this key pathway in colorectal cancer. In the legend is represented the different targeted therapeutic and drugs strategies developed in this setting of patients.

It is known that the RAS/RAF/MEK/ERK signaling cascade, also known as the mitogen-activated protein kinase (MAPK) pathway, plays an essential role in cellular proliferation, differentiation, survival, and apoptosis [35,36] (Figure 1).

The RAF protein, a serine/threonine kinase, contain three conserved regions consisting of CR1, CR2, and CR3. CR1 and CR2 are located in the N-terminus. CR1 is the main binding domain for RAS, CR2 is the regulatory domain, and CR3, which is situated in the C terminus, acts as the catalytic kinase domain. CR3 is made of two important regions for RAF activation: the activation segment and the regulatory region. The RAF family is mainly constituted of aRAF/bRAF/cRAF, where BRAF (v-RAF murine sarcoma viral oncogene homolog B; B-type RAF kinase) is the most frequently mutated, and is the most potent activator of MEK [37,38].

BRAF activating mutations, which are usually mutually exclusive with KRAS mutations, represent 5–15% of mCRC, and are associated with a poor prognosis in stage II, III, and IV [15]. A study performed in 2018 reported a median OS for wild type (wt) KRAS, NRAS, and BRAF mCRC patients of, 49.2, 36.2, 30.1, and 22.5 months, respectively [39]. The BRAF protooncogene is located in chromosome 7, and is composed of 18 exons; the typical mutation lies on a valine to glutamic acid change at codon 600 (BRAF V600E), corresponding to almost 95% of the mutations observed. This alteration, identified in up to 7% of human cancers, results in a constitutively activated protein, similarly to what happens in KRAS mutated tumors. Dual mutations of both RAF and RAS are rarely observed: in a report including a total of 2530 patients from three randomized trials in metastatic CRC (COIN, PICCOLO and FOCUS), there were only eight cases (0.3%) [40,41]. Even though BRAF and KRAS work close in the EGFR pathway, their mutations result in different gene expression patterns, with an even greater heterogeneity found among BRAF mutated mCRC, as shown in a study of 218 BRAF-V600E mutated colorectal tumors. There are two subgroups identified: one subgroup expresses high KRAS/mTOR/AKT/4EBP1/EMT activation, whilst the other one was characterized by cell-cycle dysregulation. They both were independent of microsatellite instability, PI3K mutations, sex, and sidedness. These two subgroups may correlate to the different responses to BRAF and MEK inhibitors; hence, further investigation is required to clarify this issue [36,42]. 

Phenotypically BRAF V600E mutated mCRC is associated with right-sided cancer. In the above-mentioned report, there were 22.6% of BRAF mutations in the right-side colon cancer vs. only 5.1% in the left-side colon cancer. Apart from the right-sidedness mentioned, this mutation is more related to patients aged above 70 years, females, mucinous and poorly differentiated histology, peritoneal, and nodal metastases, being less frequent in tumors with lung metastases [36,40,41].

Even though the majority of mutations are V600E, there are also rare mutations with a better prognosis, such as BRAF D594G or G596N mutant tumors, showing a median OS of 62 months, which is significantly higher than that of BRAFV600E-mutated tumors (11.4 months) and BRAF wt tumors (43 months). Those differences in survival could be explained by different alterations in BRAF regarding type of mutation. V600E is a kinase-activating mutation, in contrast with D594G or G596N, which are kinase-impairing mutations [43,44]. Furthermore, in comparison with V600E, those mutations are more frequently present in the rectum, showing a non-mucinous histology and no peritoneal dissemination. The exception, found in rarer BRAF mutations, in terms of prognosis, is codon 601/597 mutations, the behavior of which is similar to V600E mCRC. As a result of the different mechanisms involved, a classification of BRAF mutations has been proposed: class 1 (V600E) and 2 (601/597), with a similar prognosis, and class 3 (594/596), with a significantly better OS [43]. In recent studies, almost 20% of BRAF-mutated tumors were non-V600E and more frequently identified in young male patients, high grade, and right-sided primary tumors. Non-V600E tumors may be sensitive to EGFR inhibitors, in contrast with V600E tumors, but, due to the small sample size, further studies are required to definitely consider this [44,45,46,47].

BRAF mutations are frequently observed in sessile serrated adenomas, and are associated with MSI, hypermethylation, and minimal chromosomal instability. BRAF mutations are present in 40–60% of MSI mCRC, with no cases described in Lynch syndrome. There is an overlap between MSI and BRAFV600E, with an important impact on prognosis: the poor prognosis associated with the latter is compensated by the favorable effect of MSI. Nevertheless, a recent meta-analysis by Manthravadi S et al., with 1164 MSI non-metastatic CRC, showed that the presence of BRAF mutation entails worse survival, without statistically significant differences in disease recurrence [48,49,50].

From a chemotherapeutic point of view, there are no differences between BRAF mutated cancer and wt tumors regimens. Taking into account clinical characteristics in patients that harbor BRAF tumors, the preferred chemotherapeutic regimen is 5-fluorouracil, folinic acid, irinotecan, and oxaliplatin (FOLFOXIRI) + bevacizumab in metastatic disease. In adjuvant setting regimens containing capecitabine + oxaliplatin or 5-fluorouracil + oxaliplatin are being recommended, with no differences with wt tumors [36,50].

In general, patients harboring BRAF mutations develop a worse prognosis disease in comparison with BRAF wt patients. The median OS achieved by BRAF mutated patients, with standard treatment and targeted therapies, is less than 12 months compared to approximately 30 months in patients with BRAF wt tumors. Even though no differences have been observed in terms of PFS, only 33% of patients receive second line treatment, vs. more than 50%, in the case of BRAF wt tumors. [36,40].

While the impact of BRAF mutation status in prognosis is clear, the benefit when using EGFR-directed treatments remains uncertain. It is well known that KRAS activating mutations (around 40% in mCRC) trigger the RAS-RAF-ERK pathway, with a subsequent resistance to anti-EGFR therapy. A meta-analysis performed by Rowland A et al. in 2015 evaluated the effect of anti-EGFR therapy in BRAF-mutated and BRAF-wt tumors. Although, initially, the hazard ratio for PFS and OS seemed favorable to BRAF-wt tumors, further statistical analysis showed no significant differences. Another meta-analysis, published the same year by Pietrantonio F et al., showed no benefit from the use of panitumumab or cetuximab in patients with RAS wt/BRAFV600E mutant tumors, in comparison with wild type tumors [36,51,52,53,54]. 

A phase II randomized trial demonstrated that adding panitumumab to FOLFOXIRI was beneficial in terms of the response rate in BRAF-mutated mCRC, when compared with chemotherapy alone: 71% vs. 22%. However, no difference in median PFS was demonstrated (6.5 vs. 6.1 months, with and without panitumumab, respectively) [55]. 

Overall, the guidelines do not have a definitive recommendation about the use of anti-EGFR therapy in combination with chemotherapy in those patients [56].

A non-projected sub-analysis from Hurwitz et al. showed a 2-fold increase in OS, with the use of bevacizumab in BRAF-mutant mCRC (16 vs. 8 months). However, the number of patients included in this post-hoc analysis was only 10. A large US trial comparing bevacizumab with cetuximab in mCRC reported a better OS in BRAF-mutant tumors treated with bevacizumab (15 months), compared with those treated with cetuximab (11.7 months). Nonetheless, the difference was not statistically significant [57,58,59].

The combination between FOLFOXIRI and bevacizumab in metastatic CRC results in a 90% of response rate, a median PFS of 12.8 months, and an OS of 30.9 months in a post hoc analysis performed in a subgroup of 10 patients [60]. Subsequent studies have confirmed these results: 214 patients, including 15 BRAF-mutant tumors, treated with the triplet chemotherapy plus bevacizumab, obtained a PFS and OS of 9.2 and 24.1 months, respectively [61]. The TRIBE trial showed that a chemotherapy triplet is associated with a better outcome than a doublet: 16 patients with BRAF-mutant tumors treated with FOLFOXIRI + bevacizumab had a median OS of 19 months, with better outcomes, although not statistically significant, in comparison of the combination of FOLFIRI + bevacizumab (10.7 months) [62]. After all these results, FOLFOXIRI plus bevacizumab is now considered the standard of care for the first line treatment of BRAF-mutant CRC [36]. Our recommendation is to offer a clinical trial to patients with metastatic CRC BRAF mutated tumors.

## 5. New Strategies

### 5.1. Tyrosine Kinase Inhibitors

When first line treatment fails in these patients, diverse strategies have been developed. Even though not many BRAF-mutant patients are fit to receive further treatment, this mutation is not associated with inferior outcomes after first line therapy. The use of second generation BRAF inhibitors in combination regimens are likely to work better than monotherapy. 

Since BRAF-mutant cancer cells are highly dependent on MEK/ERK signaling, the combination of a BRAF inhibitor and a MEK inhibitor (double therapy) has shown a slight increased activity in comparison with either agent alone. Corcoran et al. demonstrated a reduced level of phosphorylated ERK in biopsies performed during a study of BRAFV600E-mutant mCRC treated with dabrafenib (a BRAF inhibitor) plus trametinib (a MEK inhibitor); 43 patients were enrolled with a 56% rate of stable disease, 12% partial response, and 2% complete response for more than 3 years [63]. BRAF inhibition downregulates the negative feedback signals from ERK, resulting in the activation of the EGFR pathway. This may explain the limited action of BRAF inhibitor in monotherapy in BRAF-mutant tumors, and would suggest that concomitant EGFR inhibition may overcome this resistance [64]. However, in the VE-BASKET study, the combination of vemurafenib (BRAF inhibitor) and cetuximab showed an ORR of 15% in 26 patients [65]. Other combinations under research have also demonstrated limited activity, suggesting that BRAFV600E inhibitors reactivate the EGFR signaling pathway [66]. A mechanism of resistance that is currently being evaluated converge on the formation of RAF dimers. Current RAF inhibitors block RAF monomers but not dimers. It is expected that third generation RAF inhibitors, which do inhibit RAF dimers, may shed light to the BRAF mutated mCRC management [67].

The combination of targeted therapies against EGFR and BRAF has reported, in a phase I trial, a partial response in 19 of 91 patients included (21%) and stable disease in 59 of 91 patients (65%), with an overall tumor growth control of 86%. The BEACON trial with 665 BRAF V600E mutated mCRC patients randomized in a 3-arm phase III trial to triplet therapy with encorafenib (BRAF inhibitor) plus binimetinib (MEK inhibitor) and cetuximab vs. encorafenib, plus cetuximab vs. a control arm (irinotecan/FOLFIRI + cetuximab) in patients RAS wt in a second or third line setting. The final results in the triplet combination confirmed ORR and median OS of 26% and 9.0 months, respectively, and 2% and 5.4 months, respectively, in the control arm. The doublet therapy showed a median OS of 8.4. adverse events at G3 or higher were 58%, 50%, and 61% in the triplet-, doublet- and control-arm group, respectively [68,69].

The knowledge about acquired mechanisms of resistance is not completely understood, and some possible explanations have been suggested. For example, activations of the PI3K/AKT pathway have been described in patients receiving BRAF inhibitors, in order to keep intracellular signaling via ERK [70]. Furthermore, overexpression of the hepatocyte growth factor (HGF), or its receptor c-MET, may lead resistance to BRAF inhibitors through the PI3K/AKT pathway [71]. However, this is not the only crosstalk pathway. From in vitro models, it has been described that BRAF inhibitors resulted in a feedback activation of EGFR signaling, in order to maintain ERK phosphorylation, suggesting it may be a new possible resistance pathway [72]. Additionally, the expression of aberrant spliced forms of BRAF V600E, such as BRAF V600E ΔEx, which are not sensitive to BRAF inhibitors, or truncated isoforms that continuously activate MEK/ERK signaling through dimerization, may also be involved in the resistance to BRAF inhibitors [73,74].

Moreover, resistance mechanisms due to complex genomic alterations have been described. BRAF amplifications are related to acquired resistance to BRAF and MEK inhibitors. It also seems that high BRAF mutant allele frequency may be related with high risk of primary disease refractoriness. [75] KRAS G13D amplifications have been also described in in vitro models of melanoma cells treated with MEK inhibitors [76].

The loss of Neurofibromin 1 (NF1), as a tumor suppressor that inhibits RAS, or the amplification of Cyclin D1, as a key factor in cell cycle regulation, were described in in vitro models as resistance pathways to BRAF inhibition [71].

### 5.2. Immunotherapy

BRAF mutations do not modify the response of MSI patients to immunotherapy. In general, 3–6% of stage IV CRC have a deficiency in DNA mismatch repair enzymes. Since pembrolizumab and nivolumab have shown activity against MSI mCRC, this may be a potential therapeutic option [28]. A study evaluating the role of nivolumab in monotherapy in 74 MSI patients showed an ORR of 25% in BRAF-mutant tumors, 27% in KRAS mutated, and 41% if both were wild type [27]. A combination of nivolumab with ipilimumab obtained similar results [77]. No randomized trials have been performed to compare dual checkpoint inhibitor therapy with monotherapy. However, indirect comparisons from the CheckMate 142 trial suggest that the combination may be superior. Nivolumab, pembrolizumab, and the combination of nivolumab-ipilimumab are approved by the FDA for patients with MSI or dMMR mCRC, which have progressed to other treatments [27,77]. 

## 6. Future Promising Strategies: Chemokine Receptors 

Therapeutic research in the field of BRAF-mutant CRC is currently focused on new potential targets and combinations of known targeted therapies, with the aim of overcoming MAPK pathway resistance.

**Figure 2:**
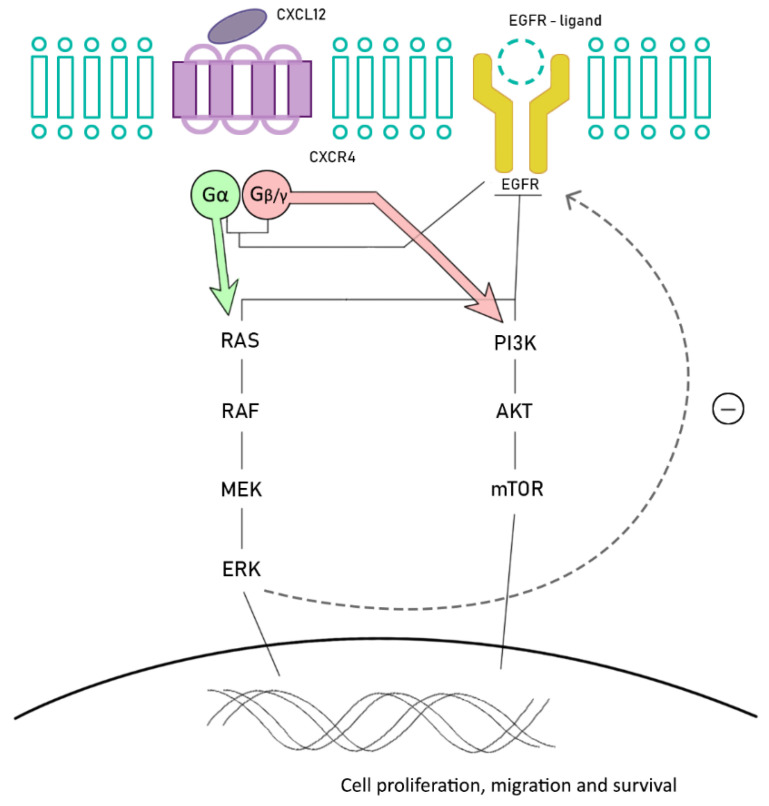
Chemokine (C-X-C motif) receptor 4 (CXCR4)/EGFR pathways crosstalk. CXCR4 is able to upregulate EGFR activation and finally ERK phosphorylation.

BRAF-mutant MSI CRC is related to the overexpression of stromal cell-derived factor-1 (SDF-1, also called CXCL12) and chemokine (C-X-C motif) receptor 4 (CXCR4), also known as fusin or CD184. CXCR4 is expressed in several cells from different organs, including colon, lung, liver, brain, and hematopoietic and progenitor cells, among others. CXCR4 belongs to a superfamily of G protein-couple receptors, and is functionally expressed in different types of cancer cells, including colorectal cancer cells. After its activation, it dissociates in two subunits: Gα, involved in regulating RAS/RAF and Gβγ, which activates PI3K/Akt/mTOR. There is a crosstalk between the EGFR and CXCL12/CXCR4 signaling pathways. CXCR4 is able to directly upregulate EGFR phosphorylation after its activation, but also indirectly, by increasing ERK phosphorylation [78,79,80,81] (Figure 2).

The CXCL12-CXCR4 axis regulates, among others, the migration and homing of lymphocytes to secondary lymphoid tissue and, also, for hematopoietic stem cells to the bone marrow. In CRC, this axis has demonstrated its role in promoting the migration, invasion (through angiogenesis), and transition of epithelial-mesenchymal tissue into neoplastic cells [78,79].

In a recent study of 78 primary CRC, CXCR4 expression was correlated with grading and response to first line chemotherapy, representing a strong and independent prognostic factor, since its high expression was correlated with a poor response in first line treatment, especially if anti-EGFR therapy was administered. A crosstalk between CXCR4 and VEGFR has been postulated by a synergistic activity from both ligands (CXCL12 and VEGF), which copes with the action of bevacizumab by restoring the angiogenesis. In fact, the high expression of CXCR4 and poor response to anti-EGFR was proven. Hence, inhibiting this axis may be a powerful strategy to deal with this resistance [81].

High CXCR4 expression is correlated with a poor histological differentiation, also being related to an increased risk of local recurrence and/or lymph node and distant metastasis in earlier stages, as well as worse OS (23 months vs. 9 months in tumors with low CXCR4 expression) [82]. 

Overall, CXCR4 may be a potential new target therapy in patients harboring BRAF mutated CRC. Moreover, thanks to the overcrossing with the EGFR pathway, CXCR4 inhibitors may overcome resistance to anti-EGFR therapy in this setting of patients. This axis has also been suggested as a potential independent prognostic factor for OS, but further studies and biology knowledge are necessary [80].

## 7. Conclusions

BRAF mutated CRC has unique clinical, morphological, and therapeutic characteristics that differentiate it from the other described molecular subtypes. The classic treatment has been based on a combination of chemotherapy treatments with anti-VEGF monoclonal antibodies. Until very recently, a tailored treatment was not available. These strategies are based on pharmacological combinations that aim to prevent cellular BRAF signaling, feedback, and its direct effectors. Furthermore, these tumors frequently develop alterations in the DNA repair proteins, and, therefore, are potential candidates for immunotherapy treatment. Although these strategies clearly benefit patients, it is necessary to continue the search for new therapies. Investigational efforts should be done in clinical and translational research, looking for new biomarkers and targets, such as chemokine receptors, to better understand the biology of the disease, to foster the development of new drugs, and to continue improving the survival and quality of life of these particular group of patients.
